# Highly efficient and automated extraction of DNA from human remains using a modified EZ1 protocol

**DOI:** 10.1080/20961790.2020.1848138

**Published:** 2021-01-18

**Authors:** Anna Barbaro, Sasha Samar, Giacomo Falcone, Angelo La Marca

**Affiliations:** Department of Forensic Genetics, Studio Indagini Mediche E Forensi (SIMEF), Reggio Calabria, Italy

**Keywords:** Forensic sciences, forensic genetics, DNA extraction, bone, tooth, EZ1 automation

## Abstract

Bones and teeth often represent the only sources of DNA available for identifying human remains. DNA in bones and teeth is generally better preserved than that in soft tissues because of the presence of hard connective tissue with a high level of calcium. Because of the extensive mineralisation, the choice of an efficient DNA extraction procedure is important to minimise the sampling of a high level of minerals and to remove polymerase chain reaction (PCR) inhibitors. Some protocols are available for DNA extraction from bones and teeth as part of the Qiagen EZ1 DNA Investigator Kit using the EZ1 Advanced XL automated purification platform. To improve the efficiency of DNA extraction from skeletal remains, the present study focuses on a modification to these already available protocols. In this study, different bones and teeth collected between 1 and 50 years after death were subjected to DNA extraction using the standard EZ1 protocol, a supplementary protocol, and a modified protocol. The modified approach included a decalcification step, whereas the Qiagen protocols worked directly on non-decalcified powder. In all three procedures, 150 mg samples were used for DNA extraction. We evaluated the quantity of DNA recovered from samples, the presence of any PCR inhibitors co-extracted, the level of DNA degradation, the quality of short tandem repeat (STR) profiles, and the reproducibility of the modified procedure. When compared with the other protocols, the modified protocol resulted in the best recovery of DNA that was free of PCR inhibitors. Additionally, the STR profiles were reliable and of high quality. In our opinion, the decalcification step increases DNA recovery by softening tissues, which allows lysis solutions to act more effectively. Furthermore, the use of two lysis solutions and the variation added to the EZ1 purification step allow for DNA recovery with quality and quantity superior to those of the previously available Qiagen-based protocols. These findings may be helpful solutions to the problems commonly encountered when dealing with difficult samples, such as bones and teeth.

**Key points**
- Bones and teeth often represent the only sources of DNA for identifying human remains.
- The choice of an efficient DNA extraction procedure is important for maximizing DNA recovery and removing PCR inhibitors.
- This study focuses on modifications to the previously available Qiagen-based protocols.
- The modified protocol enabled the best recovery of DNA, and both quality and quantity were superior to those of the previously available Qiagen-based protocols.
- The STR profiles obtained from samples extracted using the modified protocol were reliable and of high quality.

## Introduction

In some cases, bones and teeth represent the only sources of DNA for the identification of human remains. Human bodies sometimes remain exposed to the environment for days, weeks, or even years before being discovered. Environmental factors, such as UV light, humidity, and temperature, accelerate the degradation of DNA. Polymerase chain reaction (PCR) inhibitors, which are found in a variety of biological materials such as bones and teeth, may negatively affect DNA analysis and result in partial DNA profiles or complete PCR failure [1, 2].

Bone is a complex, highly organised, and specialised connective tissue with high levels of calcium. The majority of DNA in bone is located in the osteocytes. Teeth consist of enamel, dentin, cementum, and pulp tissue. Enamel is the hardest component and the most highly mineralised substance in the human body. DNA is contained in dentine and pulp. DNA in bones and teeth is generally better preserved than that in soft tissues because of the presence of hard connective tissue with a high calcium content. Because of this extensive mineralisation, the choice of an efficient DNA extraction procedure is crucial to remove PCR inhibitors and minimise the sampling of high levels of minerals [3–5].

Different DNA extraction procedures have been developed [6, 7]. Some protocols are available for DNA extraction specifically from bones/teeth using the EZ1 DNA Investigator Kit and the EZ1 Advanced XL automated purification platform (Qiagen, Hilden, Germany) [8]. This platform is designed to purify nucleic acids from a wide variety of samples [9].

All purification reagents are supplied in pre-filled EZ1 cartridges to reduce both the number of manual steps and the risk of contamination. DNA in the sample lysate is isolated in one step by binding to the silica surface of magnetic particles, after which debris is washed away. The instrument allows for barcode reading of sample tubes and reagents. It can process 1–14 samples in approximately 20 min, generating a logfile report. An internal UV light is provided for decontamination purposes. Three different DNA purification protocols (Trace, Tip Dance, and Large-Volume)^1^ are available on special EZ1 Advanced XL DNA Investigator Cards, and can be performed in conjunction with the sample pre-treatment protocols. DNA elution can be done in water or TE buffer, using elution volumes of 40, 50, 100, or 200 μL [10].

As a first step in the present study, we evaluated the quality and quantity of DNA obtained using the standard EZ1 protocol released in 2014 (indicated in the text below as QTP), as well as the supplementary EZ1 protocol released in 2016 (indicated in the text below as QSP). To increase the efficiency of DNA extraction, we then focused on developing a modified protocol (indicated in the text below as QMP).

## Materials and methods

### Bone and tooth sample preparation

Eleven different samples of bones and teeth were collected during exhumations performed in several cities in Calabria (South Italy). The time intervals after death varied (1, 5, 10, 27, 48, and 50 years). Burial conditions included coffins both in the earth and in a wall of burial niches in the cemetery. Collected samples were stored at −20 °C in the laboratory (Table 1).

**Table 1.** Analyzed bones and teeth samples.

Sample No.	Sex	Person age at death time (years old)	Time elapsed between death and samples collection (years)	Collected samples	Burial conditions
1	Male	68	1	Ilium	Earth^a^
2	Male	46	5	Humerus	Earth
3	Male	84	10	Fibula	Niche^b^
4	Female	87	10	Femur	Earth
5	Female	78	27	Femur	Niche
6	Male	83	48	Tibia	Niche
7	Male	19	50	Femur	Earth
8	Male	68	1	Incisor	Earth
9	Male	46	5	Molar	Earth
10	Female	87	10	Pre-molar	Niche
11	Male	19	50	Molar	Earth

^a^ Earth: coffins buried in the earth.

^b^ Niche: coffins buried in a wall of burial niches in the cemetery.

Bones were washed with distilled water, 80% ethanol, 5% hypochlorite, and distilled water again. The samples were left to dry, after which they were powdered in liquid nitrogen and decalcified in 0.5 mol/L EDTA for 5 to 7 days.

The teeth were cleaned with soap and then rinsed with distilled water, 80% ethanol, 5% hypochlorite, and distilled water again. Next, they were cut horizontally and the roots were ground into a fine powder. All samples were divided and then processed using the three different extraction procedures mentioned above. Although the QTP, QSP, and QMP protocols may use different volumes of sample input (Table 2), the present study used the same sample volume for all three procedures to facilitate comparisons.

**Table 2.** Comparison between the Qiagen protocols and the modified one.

Methods	EZ1 Qiagen protocol 2014 (QTP)	EZ1 Qiagen protocol supplement 2016 (QSP)	EZ1 modified protocol (QMP)
Pre-lysis treatment	Place 150–200 mg of powdered bone into a 2 mL microcentrifuge tube	Place 150 mg of powdered bone into a 2 mL microcentrifuge tube	Place 150–300 mg of decalcified fragments or powder in 500 μL lysis buffer (250 uL G2 + 250 uL ATL^a^)
	Add 600–700 μL 0.5 mol/mL EDTA (pH 8.3), and incubate at 37 °C for 24–48 h Add 20 μL Proteinase K, and incubate at 56 °C for 3 h	Add 225 μL Lysis Buffer G2 + 250 μL 0.5 mol/L EDTA (pH 8.0), + 25 μL Proteinase K, and incubate at 56 °C for 24 h	Add 20 μL Proteinase K and incubate at 56 °C at least 3 h (or until the sample is completely dissolved)
	Centrifuge at 6 000 rpm for 4 min	Centrifuge at 6 000 rpm for 4 min	Centrifuge the sample into a QIAshredder tube for 5 min at 12 000–14 000 rpm
EZ1 automated purification	Transfer 200 μL of the supernatant to an EZ1 sample tube and use “Trace Protocol”^b^ Or Transfer 500 μL of the supernatant to an EZ1 sample tube and add 400 μL Buffer MTL^a^ + 1 μL carrier RNA	Transfer the supernatant to an EZ1 sample tube and add 400 μL Buffer MTL^a^ + 50 uL 3 mol/L NaOAc^a^ (pH5.0) + 1 μL carrier RNA	Transfer the supernatant to an EZ1 sample tube and add 400 μL Buffer MTL^a^ + 1 μL carrier RNA
	Use “Large-Volume Protocol”^c^	Use “Large-Volume Protocol”^c^	Use “Large-Volume Protocol”^c^
	Choose the elution buffer (water or TE) and select the elution volume (40 uL, 50 uL, 100 uL, 200 uL)	Choose the elution buffer (water or TE) and select the elution volume (40 uL, 50 uL, 100 uL, 200 uL)	Elute sample in 40 uL or 50 uL TE buffer

^a^ Buffer ATL (cat. no. 19076), Buffer MTL (cat. no. 1023430) and sodium acetate (NaOAc) should be purchased separately.

^b^ DNA Purification (Trace Protocol). Press “START” to start protocol setup; press “1” (for Trace Protocol); choose the elution buffer (water or TE) and select the elution volume; load tubes containing digested samples into the EZ1 Biorobot; press “START” to start the purification procedure; the automated purification procedure takes 15–20 min.

^c^ DNA Purification (Large-Volume Protocol). Using this protocol, up to 500 μL of pre-treated sample can be processed; press “START” to start protocol setup; press “3” (for Large-Volume Protocol); choose the elution buffer (water or TE) and select the elution volume; add 400 μL Buffer MTL to each sample tube containing digested samples; load opened sample tubes containing Buffer MTL and digested samples into the EZ1 Biorobot; press “START” to start the purification procedure; the automated purification procedure takes 15–20 min.

### Modified DNA extraction procedure

Pre-lysis treatment: Decalcified fragments and powder of 150 mg teeth and bones were dissolved in 500 μL of lysis buffer (250 μL of G2 + 250 μL of ATL), to which 20 μL of Proteinase K was added. Samples were incubated at 56 °C for at least 3 h (or overnight until the substrate had completely dissolved). Lysis time varied depending on the type of sample processed. Samples were added to QIAshredder tubes and centrifuged for 5 min at 12 000–14 000 rpm.

EZ1 automated purification: Lysed samples were processed with the EZ1 DNA Investigator Kit using the Protocol DNA Purification (Large-Volume) provided in the EZ1 DNA Investigator Card. Specifically, in accordance with the Large-Volume protocol, 400 μL of Buffer MTL and 1 μL of carrier RNA were added to each sample tube containing the lysed samples. The tubes were then loaded into the EZ1 Advanced XL. Samples were eluted in 40–50 μL of TE buffer.

### DNA quantification and quality assessment

The quantity of DNA extracted from the samples of human remains was evaluated by real-time PCR using the Investigator Quantiplex Pro RGQ Kit with the Rotor-Gene Q Real-Time instrument (Qiagen). This kit allows for detection of human DNA by targeting the 4NS1CR locus, which is a 91 bp sequence present on several autosomes of the human genome. The male quantification target region is detected as an 81 bp fragment.

The Quantiplex Pro RGQ Kit also contains an internal amplification control that is used to test successful amplification and to identify the presence of potential PCR inhibitors. Finally, a DNA degradation control provides separate detection of both male and total human DNA degradation.

The obtained data were analysed with the Q-Rex software (Qiagen) combined with the Quant Assay Data Handling Tool. The quantity of DNA recovered, presence of PCR inhibitors, and DNA degradation status were evaluated [11, 12]. All samples were quantified in duplicate.

### DNA amplification

All samples were amplified in duplicate with the AmpFℓSTR® NGM SElect® PCR Amplification Kit (Applied Biosystems, USA), as well as with the Investigator IDplex Plus Kit (Qiagen). The reaction set-up and thermal cycling conditions were performed in accordance with the manufacturer’s suggested protocols [13–15]. The recommended quantity of DNA sample is 1 ng for NGM SElect and 0.5 ng for IDplex Plus. Positive controls were Male DNA Control 007 (0.1 ng/μL) for NGM SElect and Female DNA Control 9948 (0.1 ng/μL) for IDplex Plus. A negative control (nuclease-free water) was included in the amplification.

### Capillary electrophoresis

PCR products were separated and detected by capillary electrophoresis on an ABI 3130 Genetic Analyzer (LabX, Midland, Canada). The internal lane DNA standard LIZ 600 was used to determine the lengths of PCR products obtained using the NGM SElect Kit, whereas the internal lane DNA standard BTO 550 was used to determine the lengths of PCR products obtained using the IDplex Plus kit. Each sample was run in duplicate.

Allele assignment was carried out using the GeneMapper® ID 3.2 software (ThermoFisher Scientific, Waltham, MA, USA) by comparing the results to reference Allelic Ladders available in the abovementioned kits. Raw data were analysed with this software with the following standard conditions: analytic threshold of 150 RFU (lowered to 50 RFU in the case of low amounts of DNA), stutter peaks < 15%, and heterozygous peak height > 70%.

### Reproducibility study

To assess the reproducibility of analyses of DNA quantity and quality, as well as of DNA profiles obtained using the modified extraction protocol (QMP), all samples were extracted, quantified, and amplified by two different operators on different consecutive days. The same analytical conditions were used during replicate analysis to eliminate instrument-related variation. Quantification data and DNA profiles obtained by each operator were compared.

## Results and discussion

Skeletal remains are often the only source of DNA for identifying a human body. Some studies have been performed to evaluate techniques for bone/tooth decalcification and their effects on DNA extraction [16, 17]. The purpose of decalcification is to destroy the inorganic phase by removing calcium while avoiding damage to the organic phase. Ethylenediaminetetraacetic acid (EDTA) is a chelating agent most commonly used for decalcification. It demineralises bone and inactivates DNases by chelating cations such as Ca^2+^ and Mg^2+^. Although the procedure is time-consuming, it is highly efficient for the preservation of tissue structure [18].

In the present study, DNA was extracted from seven bones and four teeth using several Qiagen protocols, as well as a modified version of these protocols. Qiagen protocols (QTP and QSP) work directly on non-decalcified powder, whereas our modified protocol involves a decalcification treatment with EDTA and may be used on powdered samples and sample fragments.

### Quantification

Quantification data were analysed using Q-Rex software combined with the Quant Assay Data Handling Tool. Manufacturer threshold settings used for the Quality Assessment were the following: Degradation Index (DI): 10; Inhibition Index (IC): 1.

Results showed that use of the QTP protocol resulted in extraction of a very small amount of DNA (on the mean order of ∼10^−2^) that was often inhibited (IC value above 1). However, better recovery was achieved using the QSP protocol even though an IC shift above the threshold was still observed, indicating possible inhibition.

The supplemental protocol includes incubation in a lysis buffer that is not available in the standard protocol, which possibly boosts the extraction efficiency.

**Figure 1.**
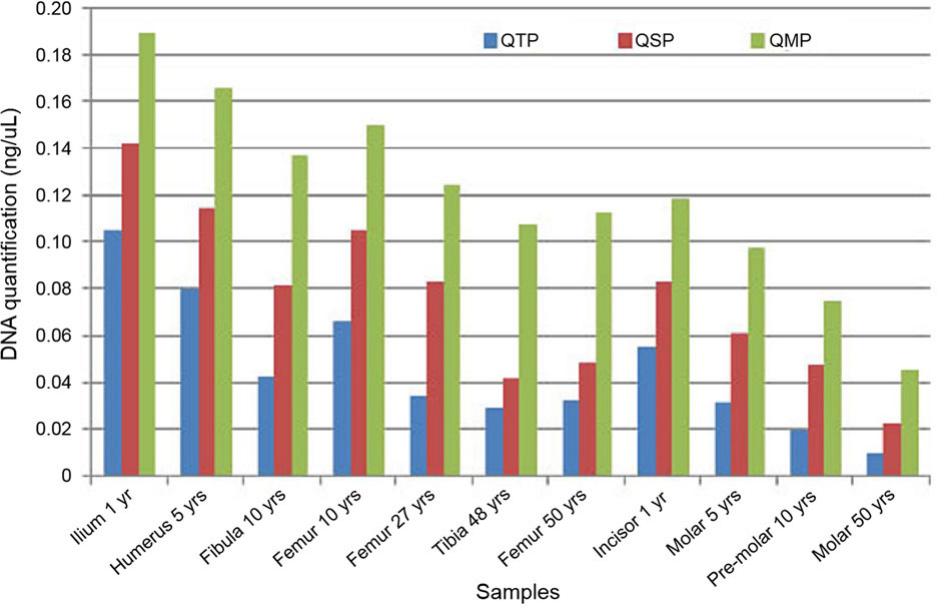
DNA quantification data for bone and tooth samples.

**Figure 2.**
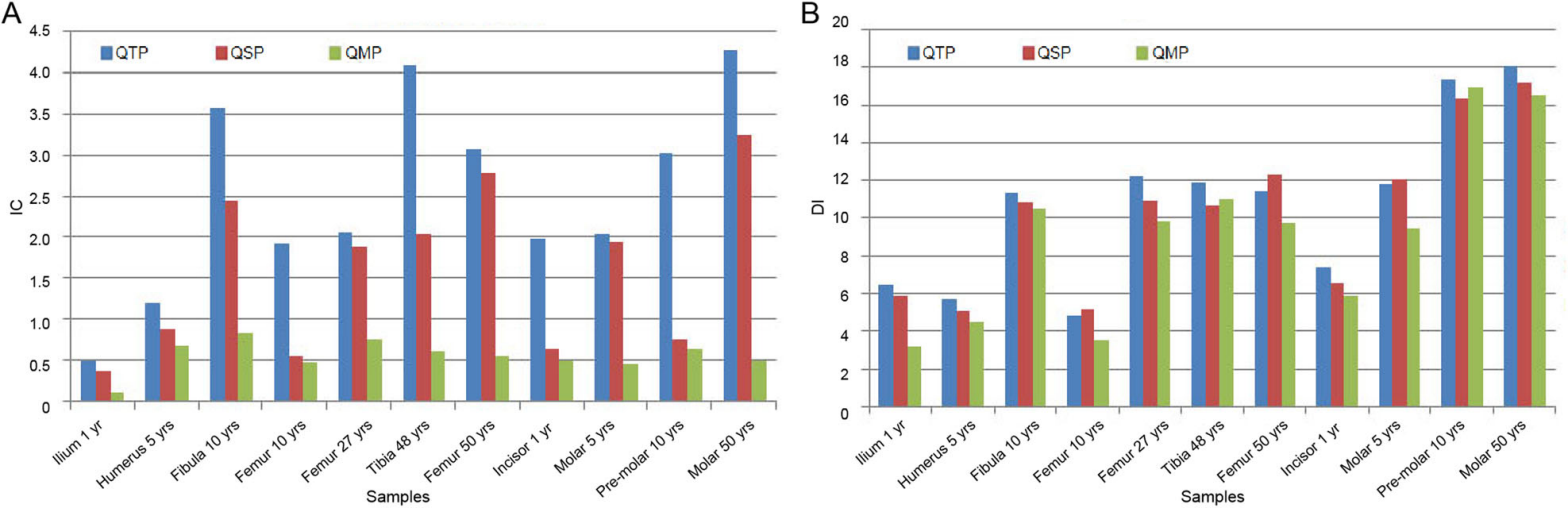
Comparison of Inhibition Index (IC) (A) and degradation Index (DI) (B) values obtained using the three protocols.

QMP achieved better recovery of DNA than the other methods: DNA recovery was 64% higher than with QTP and 39% higher than with QSP (average % across all samples). Quantification data calculated as the average per sample are reported in Figure 1. According to analysis with Q-Rex software, all samples showed IC values below the specific threshold (<1), indicating the absence of external inhibitors (Supplementary Figure S1). Additionally, the DI value was below the specific threshold (<10) in most samples, indicating that no DNA degradation had occurred. The default index value allows differentiation between DNA fragments larger or smaller than 300 bp. Full STR profiles can be obtained from DNA fragments of an average size of 300 bp. A very low quantity of DNA or an extremely high concentration of inhibitors can inhibit the amplification of the degradation target and result in a DI value above the threshold. Data of the IC and DI are plotted in Figure 2. Data are reported as the average per sample.

### Quality

We evaluated the quality of STR profiles (peak balance, preferential amplification, allelic drop-in/drop-out) that were obtained from the samples extracted by the three methods and amplified using the NGM SElect and Investigator IDPlex Plus kits. We confirmed that the DNA profiles obtained from the bones and teeth belonging to the same person showed the same genotyping results. STR profiles obtained from the bone and teeth samples extracted using QMP showed better quality than those from samples extracted with QTP or QSP. This finding was in accordance with quantification data. QMP profiles were well-balanced (heterozygous peak height > 70%), with low noise and no PCR artefacts. Peak areas and heights observed in electropherograms were relative to the amount of input DNA. The improved quality of DNA profiles observed using QMP was clearly a result of the increased DNA quantity and quality.

**Figure 3.**
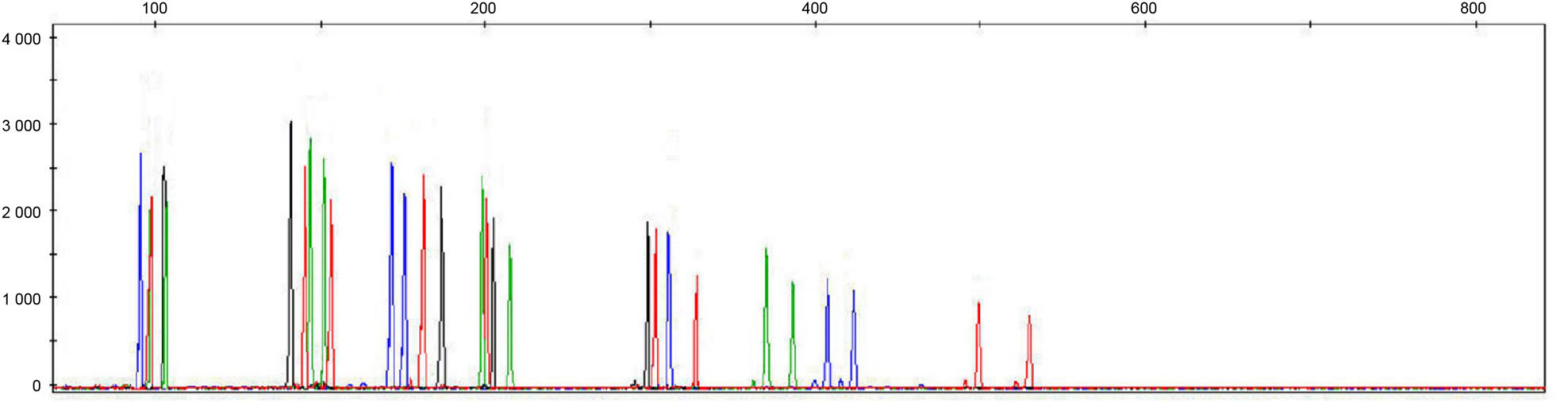
Short tandem repeat (STR) profile obtained from a 50-year-old bone sample (Sample No.: 7) extracted using modified extraction protocol (QMP) and amplified with NGM SElect.

For example, extremely reliable results were obtained from a femur belonging to a 19-year-old man exhumed 50 years after his death and buried in the earth in a cemetery (Figure 3). Anyway it is relevant to take in consideration that the age of the subject from which the remains originated, the time elapsed since death, and the location of the remains are factors that contribute to the variability in the results obtained (greater or lesser analysis success).

**Figure 4.**
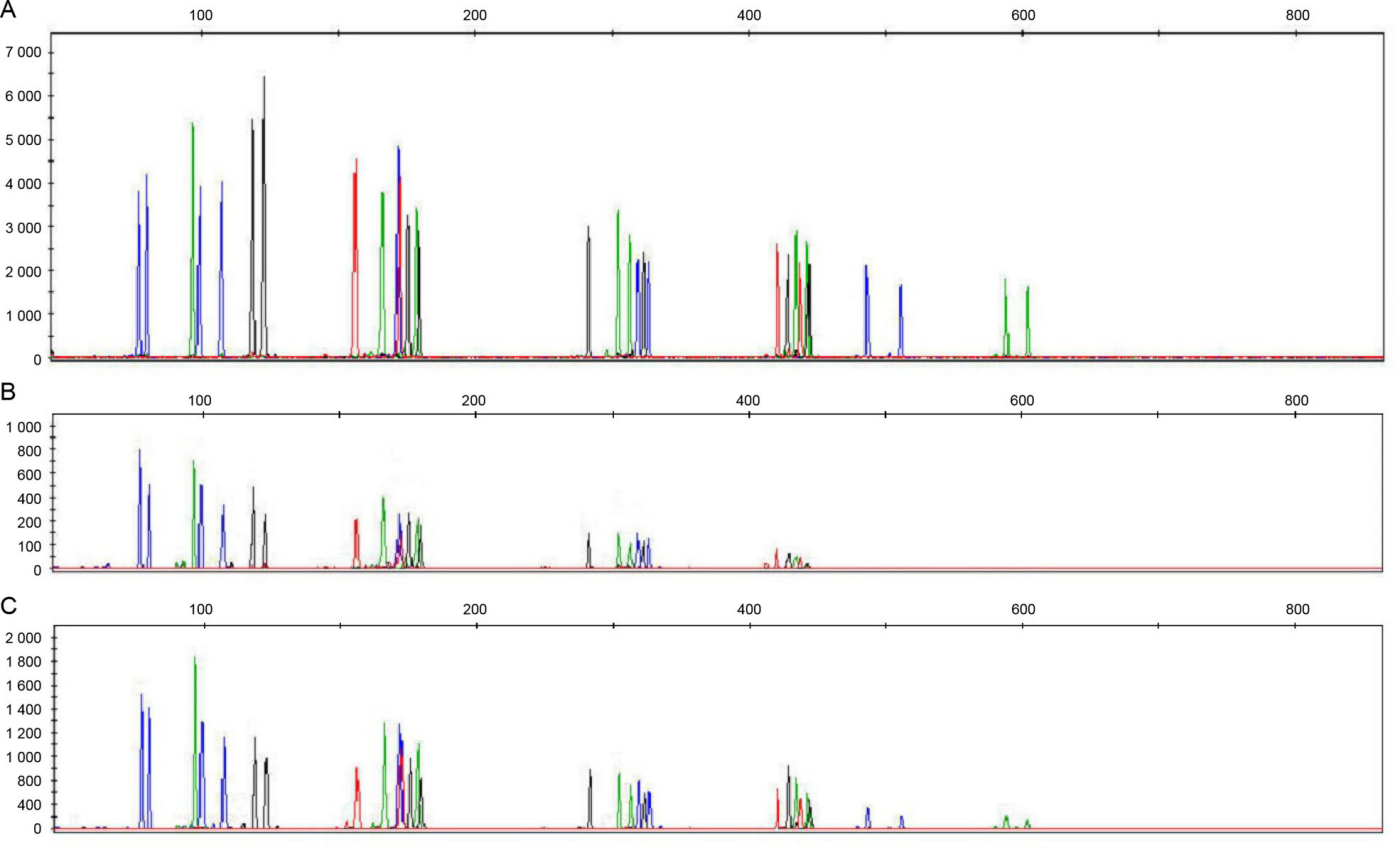
Comparison between short tandem repeat (STR) profiles (IDplex Plus Kit) obtained from a 10-year-old bone sample (Sample No.: 3) extracted using the modified extraction protocol (QMP) (A), EZ1 Qiagen protocol 2014 (QTP) (B) and EZ1 Qiagen protocol 2016 (QSP) (C).

The STR profiles obtained from samples extracted by QTP were poor, partial, and not always reliable in duplicate analysis, which was in accordance with quantification data. STR profiles from samples extracted by QSP showed a better performance than those obtained by QTP. Yet, in QSP, there was a peak imbalance, and allele or locus drop-out was occasionally observed (Figure 4).

**Figure 5.**
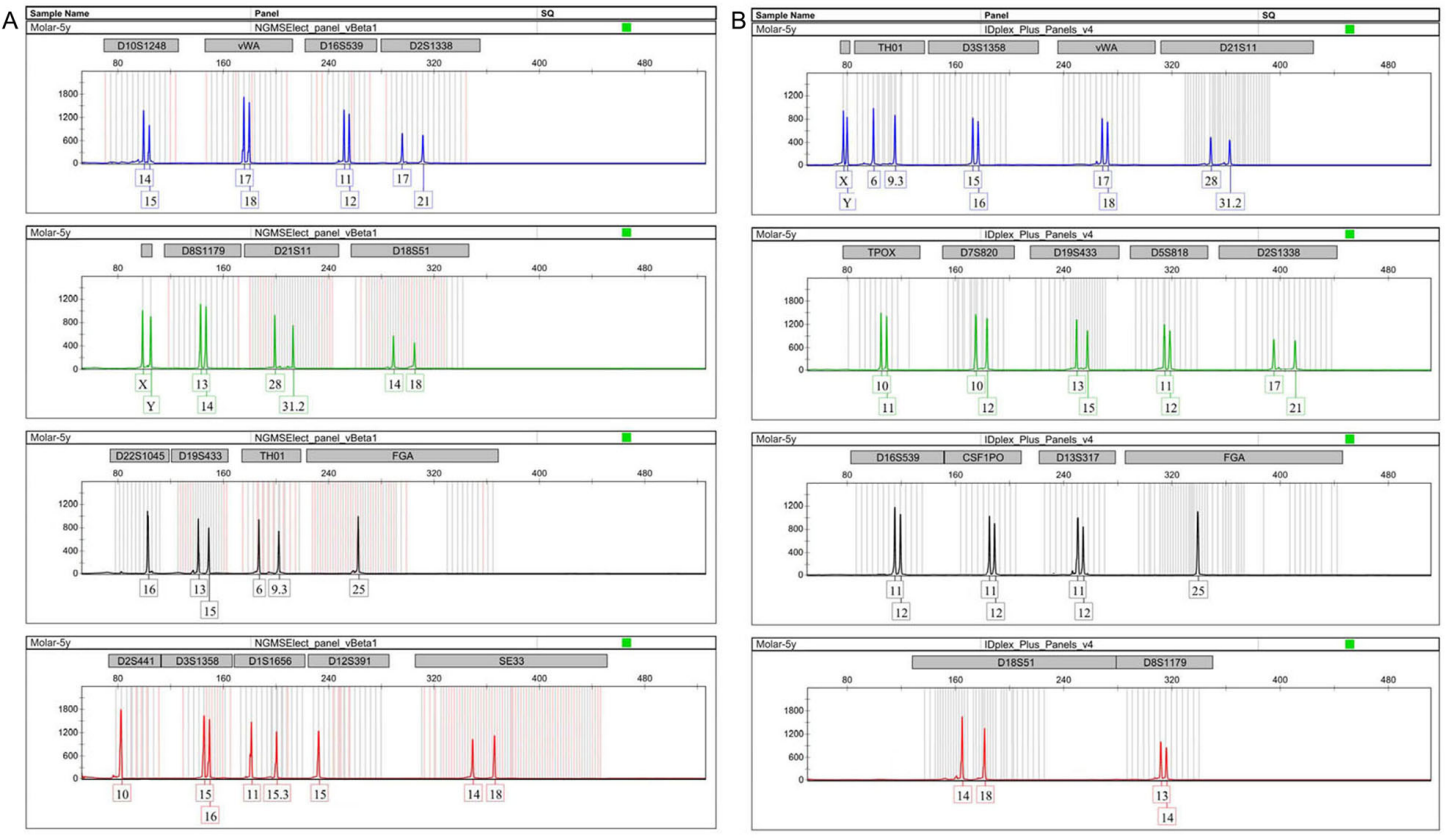
Comparison between short tandem repeat (STR) profiles obtained by two different PCR kits (NGM SElect (A) and IDplex Plus (B)) from a 5-year-old molar (Sample No.: 9) extracted using the modified extraction protocol (QMP).

Evaluation of DNA profiles obtained from the same sample extracted by QMP and amplified using IDplex Plus and NGM SElect showed that allele assignment was identical and correct for loci in common to both PCR kits (Figure 5).

**Figure 6.**
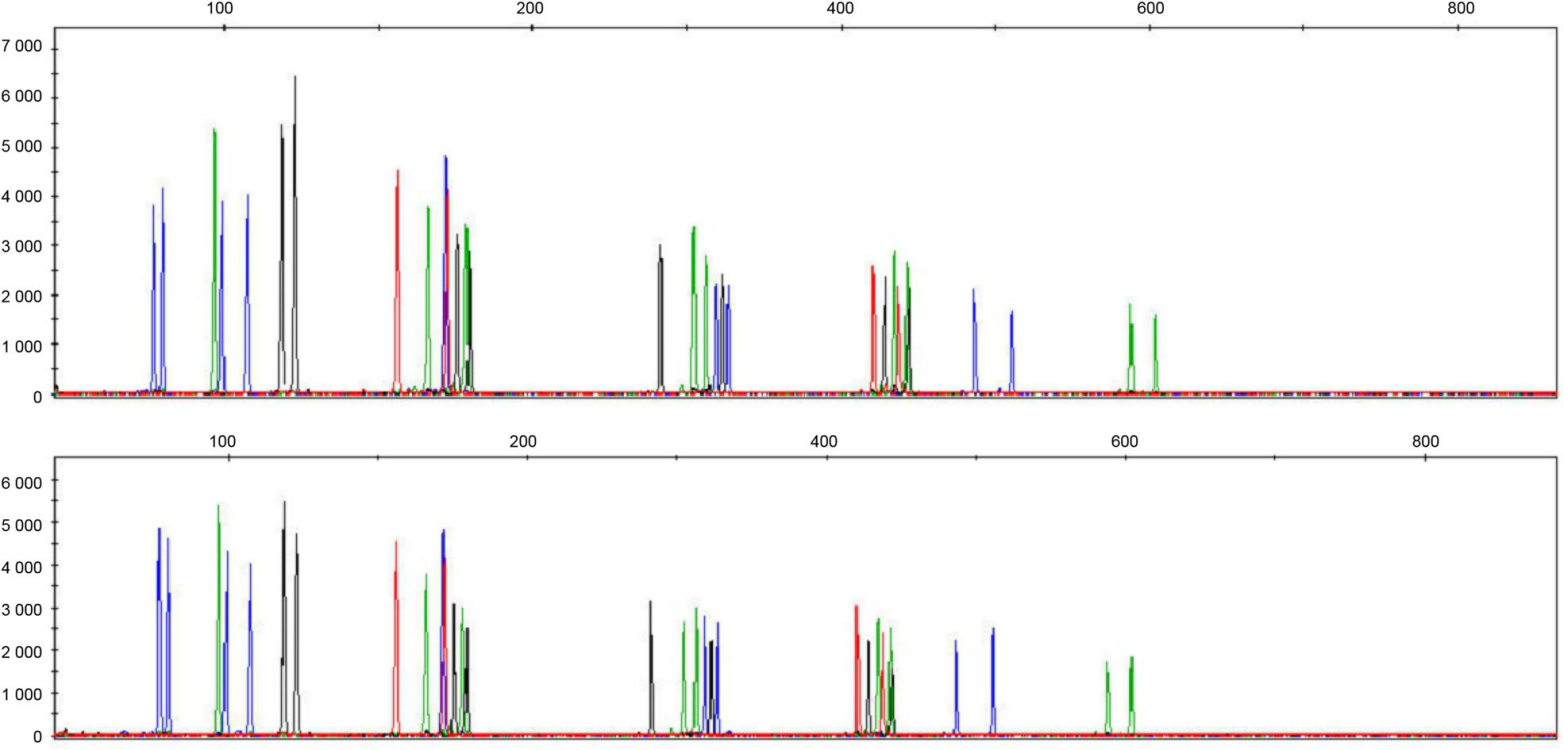
Short tandem repeat (STR) profiles (IDplex Plus Kit) obtained from a 10-year-old bone sample (Sample No.: 3) extracted using the modified protocol and amplified separately by two different operators.

Finally, a reproducibility study conducted on our modified method (described in the materials and methods section) showed that STR profiles obtained with the IDplex Plus and NGM SElect kits were reliable in a duplicate analysis performed by different operators (Figure 6). The modified DNA extraction protocol proved effective for small (150 mg bone/teeth) samples.

### Decalcification

We consider that decalcification removes minerals, softens tissues, and allows the lysis solution to act more effectively. The use of two lysis solutions (G2 + ATL) promotes cellular lysis and DNA recovery. In addition, including a supplementary purification step allows for better removal of DNA inhibitors that can interfere with PCR amplification, producing highly purified DNA.

Our findings are consistent with the conclusions of some researchers who observed benefits of total demineralisation [6, 19, 20], although Loreille et al. [16] reported a loss of DNA during each step of washing in EDTA. Nonetheless, in our opinion, the increased recovery of DNA (compared with using previous protocols) can compensate for the loss of free DNA eventually washed away during decalcification steps.

Decalcification alone is insufficient to adequately improve DNA extraction. In a previous experiment, QTP and QSP were tested using a sample pre-decalcification step (data not shown). This step improved the efficiency of the QSP protocol but did not improve the performance of QTP. This indicates the importance of choosing an efficacious lysis solution and purification treatment.

## Conclusion

In the present study, we evaluated the performance of Qiagen standard and supplementary protocols for DNA extraction from bones and teeth using the EZ1 Advanced XL and DNA Investigator Kits (Qiagen).

To increase the quantity and quality of DNA extracted from human remains, we proposed a modified version of already available protocols.

Our findings showed that the modified DNA extraction method, which includes sample decalcification with EDTA, the use of two lysis solutions, and a supplementary purification step, allows for high recovery of DNA that is free of PCR inhibitors.

The use of this effective DNA extraction method and sensitive PCR multiplexes produces highly reliable STR profiles from human bones and teeth, which are often the only pieces of evidence available for human identification in cases such as mass disasters. Our modified method has been successfully used to perform DNA typing of 50-year-old remains. The results successfully obtained in the present study confirm the potential of this method for DNA extraction from small-quantity (∼150 mg) samples. Although several protocols for DNA extraction from bones/teeth have been reported over the years, our findings are encouraging and may be useful to forensic experts dealing with the extraction of DNA from human remains. However, before applying a method in routine practice, a forensic laboratory should evaluate its cost-effectiveness in comparison with other alternatives.

## Supplementary Material

Supplemental Material

## Notes

1 The EZ1 DNA Purification “Large-Volume” protocol enables the processing of large starting volumes of up to 500 μL. This approach allows for efficient DNA purification from dilute samples with low concentrations of DNA, and also from samples that require larger volumes for lysis. The same elution volume is used as in the “Trace” protocol. The “Tip Dance” protocol differs from the “Trace” protocol because the filter tip moves back-and-forth relative to the worktable platform while pipetting.
