# Supplementary material for: Highly efficient and automated extraction of DNA from human remains using a modified EZ1 protocol
Source: Forensic Sci Res. 2021 Jan 18;6(1):59–66. doi: 10.1080/20961790.2020.1848138 (PMC8110185; doi:10.1080/20961790.2020.1848138)
Supplement: Supplemental Material [file TFSR_A_1848138_SM8544.docx]

Table 3: Bones and teeth samples quality assessment (IC and DI)

| Sample No | STP | | SSP | | SMP | |
| --- | --- | --- | --- | --- | --- | --- |
|  | IC | DI | IC | DI | IC | DI |
| 1 | Below threshold | Below threshold | Below threshold | Below threshold | Below threshold | Below threshold |
| 2 | Possible inhibition | Below threshold | Below threshold | Below threshold | Below threshold | Below threshold |
| 3 | Possible inhibition | Possible degradation | Possible inhibition | Possible degradation | Below threshold | Possible degradation |
| 4 | Possible inhibition | Below threshold | Below threshold | Below threshold | Below threshold | Below threshold |
| 5 | Possible inhibition | Possible degradation | Possible inhibition | Possible degradation | Below threshold | Below threshold |
| 6 | Possible inhibition | Possible degradation | Possible inhibition | Possible degradation | Below threshold | Possible degradation |
| 7 | Possible inhibition | Possible degradation | Possible inhibition | Possible degradation | Below threshold | Below threshold |
| 8 | Possible inhibition | Below threshold | Below threshold | Below threshold | Below threshold | Below threshold |
| 9 | Possible inhibition | Possible degradation | Possible inhibition | Possible degradation | Below threshold | Below threshold |
| 10 | Possible inhibition | Possible degradation | Below threshold | Possible degradation | Below threshold | Possible degradation |
| 11 | Possible inhibition | Possible degradation | Possible inhibition | Possible degradation | Below threshold | Possible degradation |

STP: EZ1 Qiagen protocol 2014; SSP: EZ1 Qiagen protocol Supplement 2016; SMP: EZ1 Modified protocol; IC: Inhibition Index; DI : Degradation Index.
